# Obesity as an Early Symptom of the AMIS Syndrome

**DOI:** 10.3390/jcm3041178

**Published:** 2014-10-28

**Authors:** W. Wayne Lautt, Hui Helen Wang

**Affiliations:** Department of Pharmacology and Therapeutics, Faculty of Medicine, University of Manitoba, A224-753 McDermot Avenue, Winnipeg, MB R3E 0T6, Canada; E-Mail: wlautt@cc.umanitoba.ca

**Keywords:** AMIS syndrome (Absence of Meal-induced Insulin Sensitization), Hepatic Insulin Sensitizing Substance (HISS), Meal-induced Insulin Sensitization (MIS), obesity, prediabetes, hyperinsulinemia, insulin resistance, diabetes, metabolic syndrome, hepatic parasympathetic neuropathy, rapid insulin sensitivity test (RIST)

## Abstract

We review evidence that the AMIS (Absence of Meal-induced Insulin Sensitization) syndrome describes a paradigm fundamental to development of obesity. The hypoglycemic response to a pulse of insulin is doubled after a meal as a result of Hepatic Insulin Sensitizing Substance (HISS), released from the liver to act selectively on muscle, heart and kidney. In the absence of HISS action, the hypoglycemic response to insulin is the same as in the fasted state, and only half of what it should be. Postprandial hyperglycemia ensues, with compensatory hyperinsulinemia, resultant hyperlipidemia and elevated free radical stress. Storage of nutrient energy shifts from glycogen in muscle to fat. Chronic AMIS results in adiposity, occurs with age, is accelerated with sucrose supplement, and prevented by a synergistic antioxidant. Exercise reverses AMIS, as do pharmaceuticals that mimic the “feeding signals”. The AMIS syndrome develops as a sequence of pathologies based on the consequences of absence of HISS action, including adiposity as the earliest symptom. Cardiac dysfunction, hypertension, hypercholesterolemia, and fatty liver are related to lack of HISS action. The AMIS syndrome hypothesis is mechanistic-based and accounts for the major pathologies associated with prediabetes, obesity, diabetes and metabolic syndrome. AMIS can be diagnosed, prevented and treated.

## 1. Introduction 

The objective of this article is to review what is known about Meal-induced Insulin Sensitization (MIS), the consequences of the Absence of Meal-induced Insulin Sensitization (AMIS), the AMIS syndrome, and where obesity fits into the AMIS syndrome.

We will suggest that:

- The AMIS syndrome describes a mechanistic paradigm accounting for the cluster of pathologies recognized by 13 different names including syndrome X and the metabolic syndrome.
- Obesity is the first symptom of the AMIS syndrome; other earlier signs include postprandial elevations in insulin, glucose and triglycerides.
- Whereas lack of insulin action results in type 1 diabetes, prediabetes and type 2 diabetes result from absence of a Hepatic Insulin Sensitizing Substance (HISS).
- AMIS can be diagnosed and detected. It can be prevented from deterioration associated with sugar consumption and normal aging with the use of exercise, or a synergistic balanced antioxidant cocktail.
- AMIS can be treated with one dose of drugs that mimic the two permissive “feeding signals”, when administered prior to a meal.

MIS was reported in 1998 [1,2] and an overview of the working HISS hypothesis was first reviewed in 1999 [3]. To our knowledge, only the Lautt lab in Canada, the Macedo team in Portugal, and Szilvassy’s group in Hungary have studied meal-induced insulin sensitization (MIS). The discussion is necessarily reliant on data from these laboratories. Reviews [4,5,6,7,8] follow progress in concepts. Ting and Lautt [9] reviewed the effects of adult and fetal alcohol exposure on MIS of the mother and adult offspring. Chowdhury* et al.* [10] reviewed the effect and interactions of diet, exercise and antioxidant supplementation on the AMIS syndrome and HISS action. Macedo* et al.* 2013 [11] provided an excellent overview of the role of the parasympathetic nerves in metabolic regulation in health and disease.

## 2. Meal-Induced Insulin Sensitization (MIS) 

MIS is illustrated by the dramatic increase in the glucose disposal response to insulin immediately after a meal. The concept of MIS is derived from the observation that the dynamic response to insulin, determined after a 24 hours fast, is at least doubled when tested 100 minutes after administration of a mixed meal in conscious rats [12,13], and humans [14,15,16]. This greatly amplified response to insulin results through the action of a putative hormone, hepatic insulin sensitizing substance (HISS). HISS is released from the liver in response to a pulse of insulin, and stimulates glucose uptake in the skeletal muscle, heart, and kidneys, but not in liver or adipose tissues [17,18,19]. 

### Quantifying MIS

Endogenous insulin release is pulsatile in humans [20] and animals [21,22]. Insulin is released in pulses of oscillations of ~13 minutes in both humans and rats [20,21] and 9 minutes in monkeys [22]. Increased insulin secretion is associated with an increase in magnitude, not period. Non-pulsatile administration is nonphysiological and results in a linear duration-dependent inhibition of HISS action beginning at infusion duration of 10 minutes, with insignificant HISS action remaining after 1 hour of constant infusion [23]. 

**Figure 1:**
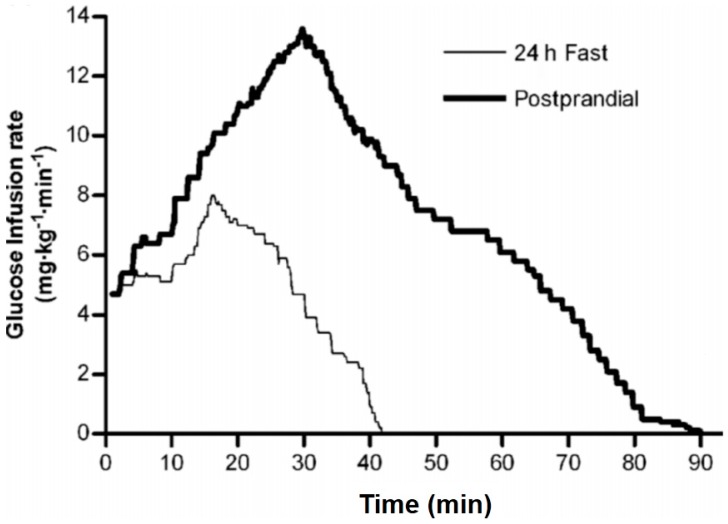
Rapid Insulin Sensitivity Test (RIST) in fasted and fed humans. The rate of glucose infusion required to maintain euglycemia is shown for healthy male volunteers after administration of a bolus of 50 mU/kg insulin in the 24 hours-fasted state. The mean RIST curves were obtained by averaging glucose infusion rates at 0.1 minutes intervals. The RIST was repeated 100 minutes after consumption of a mixed test meal. The RIST index increased from 215.5 ± 20.8 mg/kg to 681.2 ± 60.9 mg/kg due to the action of HISS, which is shown in Figure 2 ([15], © Canadian Science Publishing or its licensors).

**Figure 2:**
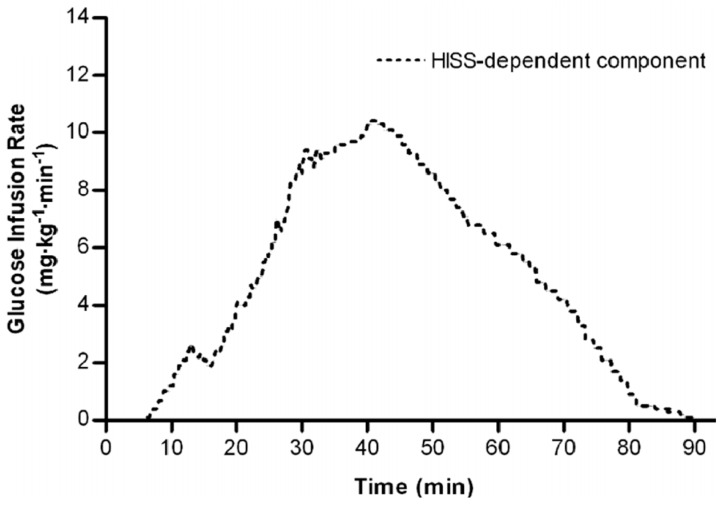
HISS-dependent insulin action in humans. Dynamic HISS action is calculated from the difference between the RIST profiles in the fasted and fed state from Figure 1. The RIST reveals a distinctive pulsatile HISS action beginning 6.3 minutes after insulin administration and continuing for 48 minutes beyond the direct action of insulin ([15], © Canadian Science Publishing or its licensors).

The dynamic action of insulin and HISS can be determined using the insulin tolerance test [24]; however, the ensuing hypoglycemia limits its use. To avoid the hypoglycemia, and to detect the dynamic response to pulses of insulin, the Rapid Insulin Sensitivity Test (RIST) was developed and has been used in cats [25], anesthetized [2] or conscious [12] rats, mice [26] rabbits [27] and humans [14]. The RIST is a rapidly sampled and adjusted euglycemic clamp that provides reproducible test scores up to 4 consecutive times in anesthetized rats [2]. An operating procedure is described for the RIST in animals [2] and humans [15]. The RIST index is the amount of glucose that was required to be infused to compensate for glucose uptake while maintaining a constant arterial glucose level. The response to a pulse of insulin is increased by at least 100% after a meal in rats [2,12] and 200% in humans ([15], Figure 1 and Figure 2). 

**Figure 3:**
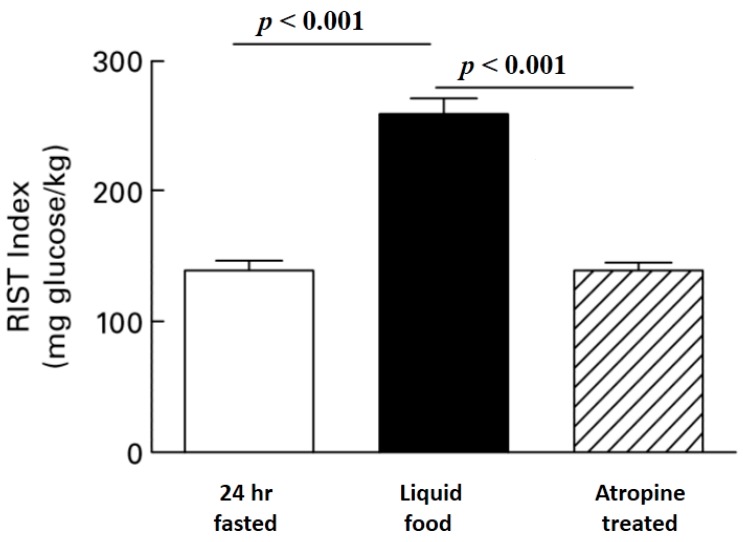
Atropine reverses Meal Induced Insulin Sensitization (MIS). The RIST was determined in 24 hours fasted unrestrained rats. Food was injected via a surgically implanted gastric catheter and the RIST was repeated after glucose levels were stable. Atropine completely reversed meal-induced insulin sensitization ([13], reprinted with permission).

**Figure 4:**
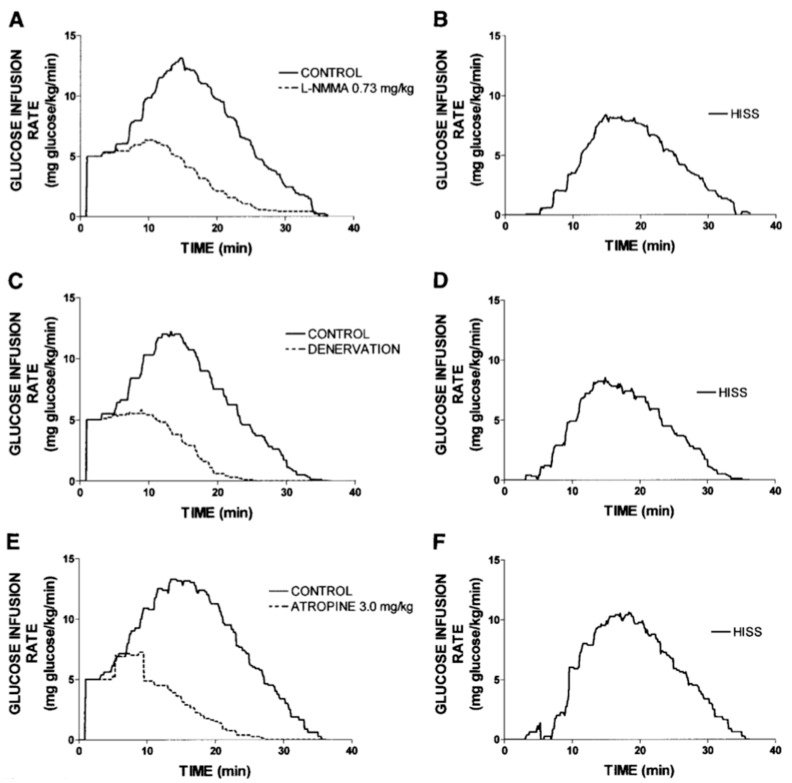
HISS action revealed by blocking the parasympathetic feeding signal. The dynamic insulin action using the RIST in fed, anesthetized rats shows a normal postprandial control RIST that is reduced by 56% by eliminating the hepatic parasympathetic feeding signal to block HISS release. Complete reversal of MIS results from blocking hepatic nitric oxide synthase, blocking muscarinic receptors using atropine, or physically interrupting the signal by surgical ablation. HISS action is revealed by subtracting the blocked profile from the control fed profile as shown in the right side column. HISS action commenced 3.1 minutes after onset of insulin action and continued for 9–10 minutes after completion of HISS-independent (direct) insulin action [28].

A more efficient method to differentiate HISS from insulin action is to carry out the studies in fed, anesthetized animals when HISS action is maximal, and then again after HISS action has been blocked by any of several means (e.g., Figure 3). To quantify MIS, two RISTs are performed for each rat. The first is done in a stable control state in fed animals. The second RIST is performed after blockade of HISS release with intravenous atropine and reestablishment of a stable glycemic baseline. The first RIST index includes the effects of both HISS-dependent and HISS-independent components, and the second RIST index represents only the HISS-independent component. The hepatic parasympathetic nerve signal can be eliminated by blocking cholinergic receptors, blocking nitric oxide synthase or surgical denervation (Figure 4).

## 3. Atropine Blockade of HISS Release 

The use of atropine as a tool to differentiate HISS and insulin action has been controversial because of the conclusion that atropine at appropriate dose blocks MIS without having other major metabolic consequences relevant to the acute dynamic response to a pulse of insulin. The use of atropine is demonstrated in Figure 3 where the RIST index in conscious unrestrained rats increased from a 24 hours fasted level of 138.8 (SE 8.9) mg glucose/kg body weight to 259.0 (SE 13.8) mg glucose/kg when measured 90 min after gastric injection of a test meal and at a stable glycemic baseline. Atropine administration inhibited the RIST index to 139.2 (SE 6.9) mg/kg while insulin and glucose levels remained at postprandial levels [13]. The profile of insulin concentration after bolus administration was not different in fed subjects before and after atropine [15]. Atropine has no effect on insulin action in fasted rats, where physiological suppression of HISS release already exists [5,29]. Similarly, if hepatic nerves are cut to eliminate the permissive neural feeding signal, HISS release is blocked in the fed state, and the remaining direct response to insulin is not altered by atropine, and is not different from the fasting response [13]. Atropine has no effect on the RIST index if HISS release has already been blocked by dietary sucrose [13,30] or fat supplementation [31] or stress [32]. 

## 4. HISS Action Related to Duration of Fasting

The ability of insulin to cause HISS release is maximal by the time glucose levels stabilize, after about 90 min when a valid RIST can be carried out. The RIST is less in anesthetized rats, but is reproducible for up to 4 consecutive tests over a 4–5 hours period [2]. The conscious rat has the same degree of MIS after voluntary consumption of chow or injection of liquid food into the stomach [12,13]. Although the MIS is quantitatively larger when food is introduced in the conscious state, it declines in the conscious state by about 10% per hour [12]. By 6 hours the HISS action is reduced by about 50% and by 75% after 18 hours [24,28]. By 24 hours of fasting, insulin no longer causes a significant release of HISS. Thus, HISS action is physiologically suppressed in the fasted state where the hypoglycemic actions of insulin are not required or appropriate. Observations in the fully fasted state are made in the absence of HISS action.

## 5. The Feeding Signals 

**Figure 5:**
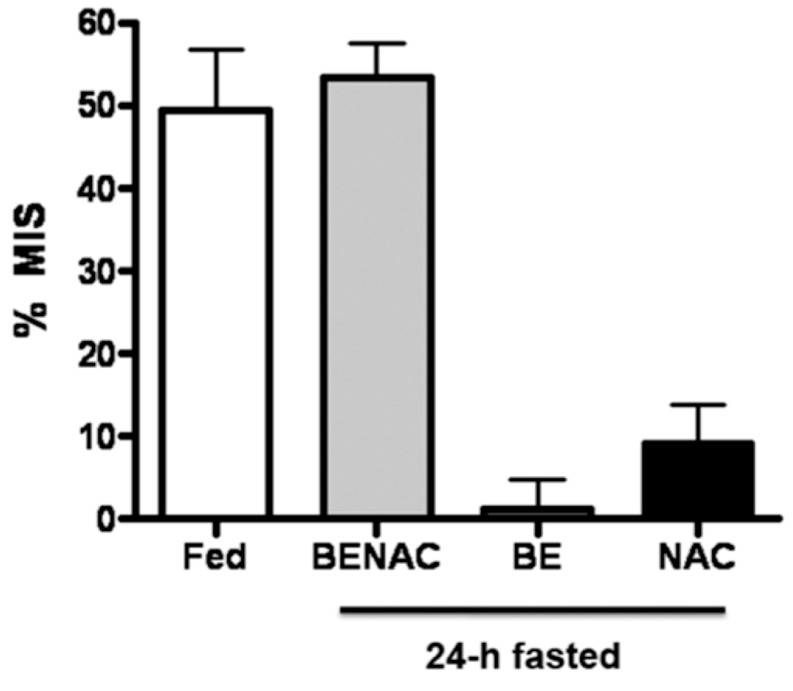
MIS induced by mimicking the two feeding signals administered in the fasted state. Mimicking the two feeding signals by drug administration in 24 hours fasted rats results in the same degree of meal-induced insulin sensitization (MIS), as produced in response to the liquid meal. The administration of a single compound (BE, bethanechol; NAC, *N*-acetylcysteine) resulted in no significant insulin sensitization, thereby demonstrating the synergy of the dual therapy (BENAC). These data are consistent with the hypothesis that two background permissive feeding signals to the liver are required to allow insulin to stimulate HISS release and that the feeding signals result in insulin sensitization similarly whether the signals are delivered by a meal or by pharmaceuticals ([37], © Canadian Science Publishing or its licensors).

**Figure 6:**
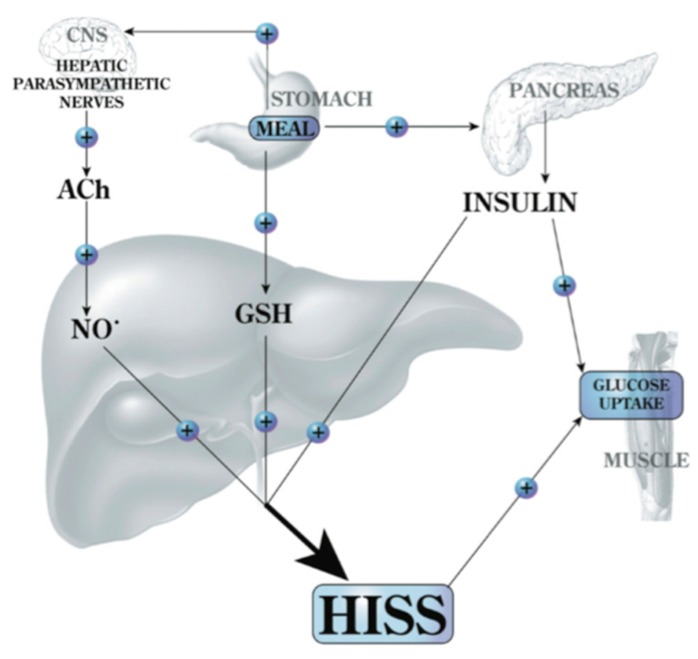
Feeding signals for HISS release. Feeding results in an increase of hepatic glutathione (GSH) and a parasympathetic signal to the liver that acts, via acetylcholine (ACh), on muscarinic receptors to activate NO release. Both of these signals are permissive and both are needed in order for insulin to cause the release of HISS. HISS acts selectively on skeletal muscle (recently also shown to act on heart and kidney [19]). Blockade of any portion of these pathways leads to blockade of HISS release and a state of HISS-dependent insulin resistance. HISS release is physiologically regulated to be absent in the fasted state, but when HISS release is not activated by feeding, its absence is suggested to account for postprandial hyperglycemia, hyperinsulinemia, hyperlipidemia, and increased oxidative stress. Chronic lack of HISS action results in a progressive and predictable series of homeostatic dysfunctions typical of type 2 diabetes ([8], © Canadian Science Publishing or its licensors).

For a current overview of the “feeding signals” see Macedo* et al.* 2013 [11]. MIS results when food in the upper gastrointestinal tract causes two permissive, synergistic feeding signals to be delivered to the liver, thereby allowing pulses of insulin to stimulate HISS release. One signal is delivered via hepatic parasympathetic nerves acting on muscarinic receptors, and subsequent activation of nitric oxide synthase [33,34] and elevated cGMP. The second is a chemical signal that is seen as an approximately 40% elevation in hepatic glutathione (GSH) [35,36,37]. In the presence of these 2 feeding signals administered to fasted rats, insulin causes the release of HISS (Figure 5) [37]. Either signal alone is insufficient to activate the HISS pathway [37,38], Figure 6 [8].

### 5.1. The Insulin Signal

To elicit maximal HISS release in response to insulin, the dose must be administered over a duration of less than 10 minutes. A 30 seconds or 5 minutes administration of 50 mU/kg resulted in similar dynamic responses, but infusions over a longer period resulted in a linear duration-dependent suppression of HISS release that was complete by 1 hour [23,24]. HISS action is linearly related to the dose of insulin with ~55% of the postprandial response to insulin being HISS-dependent at doses from 10–200 mU/kg. Higher doses and longer infusion times suppressed HISS release in subsequent tests.

Maximal HISS release in response to insulin appears to occur only if the pulsatile response returns to baseline between pulses of administered insulin (personal observation). The background steady state insulin concentration does not affect the response to pulses, as shown by similar RIST indexes in fasted rats during intravenous or intraportal infusions that raised background insulin levels 8 fold [39]. 

### 5.2. The Sensory Signal

Injection of a liquid meal into the stomach of conscious rats resulted in the same degree of MIS as seen with voluntary consumption of chow, showing that pre-gastric feeding signals are not involved [13]. Sucrose or glucose does not activate MIS [13]. 

Cholycystokinin (CCK) has been suggested to provide a role in the afferent signal transmission. Proglumide, a CCK inhibitor, produced dose related suppression of the RIST in fed, but not fasted rats [40] or in fed rats with CCK receptor deficiency [41]. It has been suggested that vagal afferent nerves in the gut are activated by food constituents and result in serotonin-dependent vagal reflexes [42]. Regardless of the afferent signal mechanism, provision of the two feeding signals directly to the liver can result in a degree of insulin sensitization equal to that produced by a meal [37,38]. 

## 6. Does HISS Exist? 

Referees of journals and grant committees have often been brutally skeptical about the new paradigm suggested by the HISS hypothesis, primarily because we do not have a chemical identification of HISS. Although the molecular structure is not known, much is known about its regulation, actions and roles. It has been shown in carnivores, herbivores, omnivores and humans. A diagnostic, a preventative and a therapeutic have been developed based on these concepts. Regardless of whether we have extended our studies from* in vivo* homeostatic focus to complex chemistry, the existence of MIS is proven. MIS is dependent on some “hormone” and the actions are clearly differentiated from those of insulin. We have suggested the tentative name of HISS because the original observations indicated that cutting the hepatic nerves resulted in a reduced sensitivity to an insulin tolerance test [43] and the effect was across the hindlimbs, not the liver [17], thus the term Hepatic Insulin Sensitizing Substance (HISS).

HISS release in the fed state is blocked by hepatic denervation, as shown by reduced metabolic and vascular responses across the hindlimbs [44]. Continuous intraportal infusion of ACh mimicked the background permissive nerve signal and restored the ability of insulin to stimulate HISS release, increasing both the metabolic and vascular responses of the hindlimb. The intraportal dose of ACh is rapidly metabolized and did not recirculate to cause direct effect on the hindlimbs, as baseline glucose and blood flow remained constant. The same dose of ACh given intravenously is rapidly metabolized and did not restore HISS release from the liver, and also had no direct effect on the hindlimbs. Intraportal ACh acted on the denervated liver to restore the metabolic response [18,45] and hindlimb vasodilation [44]. Similar restoration of the nerve signal is shown for nitric oxide donors [33,36]. Some substance is released by the liver in response to insulin and has metabolic, and vascular effects in the hindlimb. MIS was recently also shown to act on the heart and kidneys [19]. 

The permissive nature of the signal is shown by the lack of baseline effect on glucose or blood pressure but full blockade of MIS by hepatic denervation and restoration to predenervation levels by continuous infusion of the neural mimic, whether cholinergic, or nitrergic. Providing the two feeding signals in the fasted state results in no baseline changes but restores the ability of insulin to stimulate HISS release (Figure 5).

## 7. Absence of Meal-Induced Insulin Sensitization (AMIS)

AMIS results from absence of (or diminished) HISS action following a meal. A healthy MIS involves the action of insulin as well as the action of HISS, with the result being tight control of glucose levels and storage of nutrient energy primarily as glycogen in muscle. Acute absence of HISS action results in postprandial hyperglycemia, compensatory hyperinsulinemia and resultant hyperlipidemia and free radical stress and a shift in nutrient energy storage to fat for each meal after which AMIS occurs. Each of these conditions has been independently linked to cardiovascular disease (reviewed by Lautt 2007, [7]). HISS also accounts for the vasodilation in skeletal muscle associated with insulin, and AMIS is associated with lack of a dilator response to insulin administration [44]. The consequences of chronic AMIS are predictable and progressively lead to obesity at an early stage of the AMIS syndrome, and later to resistance to the direct action of insulin.

There is a strong relationship between obesity and the incidence of diabetes [46]. Fat accumulates mainly in subcutaneous adipocytes, but deposition is also found in ectopic sites such as abdominal viscera, liver, muscle, heart, and pancreas [47]. Visceral fat represents approximately 10%–15% of total body fat [47], and there is a strong correlation not only between visceral fat and whole body adiposity [48] but also between visceral fat and ectopic fat deposition of various organs like liver [49,50], heart [51,52], and muscle [47].

There are two opposing paradigms that provide explanation for the possible links between insulin resistance and obesity. It has been suggested that fat accumulation in tissues causes insulin resistance. One version is that obesity causes an increase in visceral fat mass that secretes free fatty acids and inflammatory cytokines into the hepatic portal vein, leading to peripheral insulin resistance [53,54,55,56]. Fat accumulation in skeletal muscle has also been suggested as the cause of insulin resistance in skeletal muscle [56]. Conversely we suggest that the failure of postprandial HISS release causes hyperglycemia and hyperinsulinemia, leading to a shift in the nutrient storage from glycogen to fat and resulting in progressive adiposity [3,8]. Fat accumulation in tissues is secondary to AMIS.

Ribeiro* et al.* [57] first showed that a sucrose supplemented diet resulted in severe reduction of HISS action over two weeks. By six weeks, body weight was not increased over the control group, but by nine weeks weight had begun to accumulate. In the same nine week sucrose model, AMIS can be reversed by pre-meal provision of drugs that mimic the two feeding signals to the liver (Figure 5, Bethanechol mimics the nerve signal and *N*-acetyl cysteine elevates hepatic GSH to postprandial levels), showing that the accumulated fat does not prevent a response to HISS if the proper signals are received. The tissues remain responsive to HISS.

## 8. Chronology of Obesity in the AMIS Syndrome

**Figure 7:**
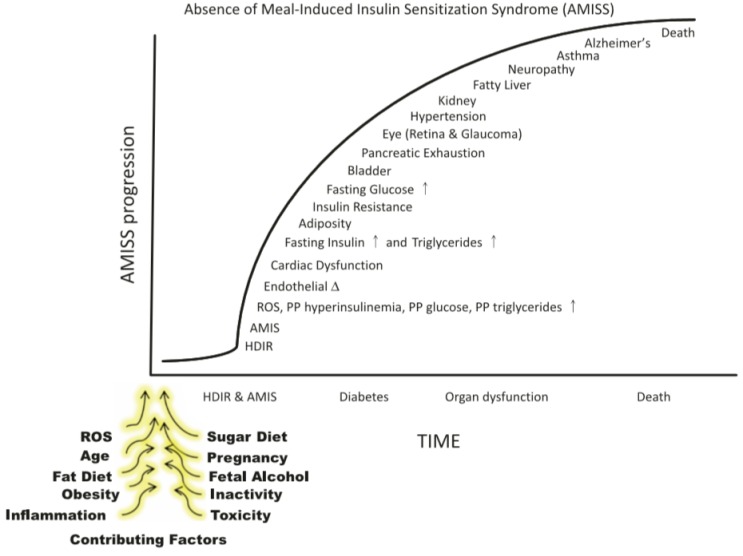
Tentative chronology of AMIS symptoms. The absence of meal-induced insulin sensitization (AMIS) syndrome describes the progressive accumulation of signs and symptoms of homeostatic disturbances caused by chronic or cumulative AMIS after each meal. HISS-dependent insulin resistance (HDIR) in the fasted state is appropriate, but if not rapidly reversed after a meal, leads to postprandial increases in glucose, insulin, lipids, and reactive oxygen species (ROS). The disease progresses to dysfunctions in major organ systems. The specific order of appearance of dysfunctions is an estimate that requires verification. The contributing factors that block HISS release and result in AMIS have been reviewed [4,5] ([8], © Canadian Science Publishing or its licensors).

To rigorously test the hypothesis that AMIS is a cause of obesity, we combined several chronic protocols, each known to have a predictable effect on HISS action. The approach used was to produce chronic graded degrees of impairment of meal-induced insulin sensitization to determine whether the degree of HISS action correlated with the predicted dysfunctions. We evaluated HISS action and a number of metabolic parameters in response to age [58,59], a chronic sucrose supplement to potentiate AMIS [57], and an antioxidant cocktail to attenuate AMIS [59,60]. By both hastening and attenuating the rate of decline in meal-induced insulin sensitization, the intention was to produce a sufficient range of dysfunctions to permit interpretable correlations and to test predictions of the AMIS syndrome and the HISS hypothesis (Figure 7).

**Figure 8:**
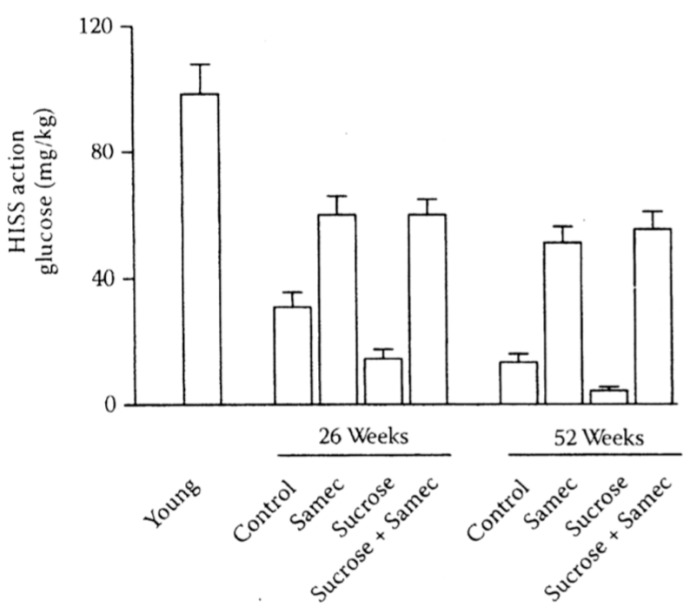
Interaction of age, sucrose, and antioxidants on HISS action. HISS-dependent insulin action assessed from the RIST index decreases with age in rats (young refers to 9 weeks of age) and is exacerbated by a low-dose (5%) sucrose supplement. The antioxidant cocktail, Samec, attenuates the decline in HISS action resulting from age and completely prevents the impact of sucrose. Results were recalculated from the pooled data of Lautt* et al.* 2008 [59] and Ming* et al.* 2009 [48,61].

**Figure 9:**
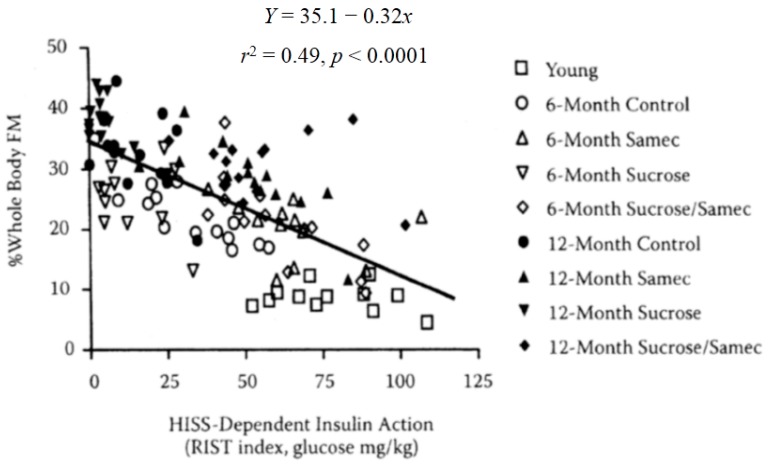
Adiposity can be predicted from HISS action, shown by pooled data from normal aging rats (9 weeks, 6 months, and 12 months), age plus sucrose, age plus antioxidant (Samec), and age plus sucrose plus antioxidant (Samec). Whole-body fat mass (FM) was estimated from bioelectrical impedance but the relationship is similar for adiposity determined by measuring regional adiposity on the basis of weighed fat mass from perinephric, perienteric, and epididymal fat pads. As HISS action decreases, adiposity increases. Results were recalculated from data in Lautt* et al.* 2008 [59] and Ming* et al.* 2009 [48,61].

Adiposity can be predicted from HISS action, shown by pooled data from normal aging rats (9 weeks, 6 months, and 12 months), age plus sucrose, age plus antioxidant (Samec), and age plus sucrose plus antioxidant (Samec, [61], Figure 8). As HISS action decreases, adiposity increases. To assess the impact on adiposity, we estimated total body fat mass and percent fat mass based on the electrical impedance method of Hall* et al.* [62]. In addition, fat mass was assessed from the weight of fat from three identifiable locations. Epididymal fat and perirenal fat do not drain into the splanchnic portal vein, whereas perienteric fat mass drains directly into the portal vein and thus to the liver. These data expressed either as total fat or percent of total body mass for each compartment show no suggestion for selective roles for regional adiposity. Total fat mass in all areas increased dramatically by 6 months and further by 12 months of age. Rats with high levels of HISS action showed low adiposity. Adiposity progressively increased with the decline in HISS action (*r*^2^ = 0.75). The relationship is similar if the data are expressed based upon the combined or individual weighed fat depots where HISS action correlates negatively with fat pad total mass in healthy (*r*^2^ = 0.77) and sucrose fed rats (*r*^2^ = 0.78). Combining the data from 9 groups with varying degrees of HISS action demonstrates the relationship to obesity (Figure 9). Age up to 1 year reduced HISS action, and sucrose hastened the dysfunction, whereas an antioxidant cocktail protected against the development of AMIS that occurs with age, and prevented most of the negative impact of the sucrose supplement. HISS action strongly correlated with adiposity in all groups. Postprandial triglycerides and insulin followed the pattern predicted by the AMIS syndrome, both increasing with decreased HISS action, and decreasing when HISS action is protected. 

Rats made obese by a high fat diet also showed a high negative correlation with HISS action and each fat pad; the perinephric fat pad showing the most sensitive index (*r*^2^ = 0.87), perienteric (*r*^2^ = 0.67), and epididymal (*r*^2^ = 0.73). A high fat diet resulted in greatly accelerated HISS-dependent insulin resistance (HDIR) and adiposity, showing no regional differentiation [63]. The high fat diet resulted in insulin resistance almost entirely accounted for by HDIR and resultant AMIS. AMIS is strongly associated with adiposity regardless of the specific index of adiposity chosen. As pertains to the view that intraperitoneal adiposity is more related to diabetes, the ratio of total fat mass to weighed fat pad mass did not change significantly as the total adiposity increased dramatically. The total weighed fat mass accounts for approximately one-third of the total body fat mass under all tested conditions. Therefore, no selective role for major regional adiposities can be supported from these data. 

## 9. AMIS Models 

HISS release can be blocked by surgical or chemical interventions, by liver damage [60,64], by stress and by alcohol in both the mother and offspring [9]. A diet high in fat [63] or sucrose [57], or a period of fasting all result in suppression of HISS release. These models of AMIS involve one or both of the feeding signals (Figure 7). Regardless of the cause, AMIS follows a progression of pathologies. 

Other means of blocking HISS action are reported, for which the specific target in the regulatory path for HISS release remains unknown. Acute stress causes somatostatin-dependent AMIS [32]. Somatostatin [65], proglumide (CCK antagonist, [40]) and indomethacin [66] inhibit HISS release. The direct, HISS-independent response to a pulse of insulin was either not altered or showed only minor effects in all of these models.

Chronic bile duct ligation led to AMIS that was reversed by acetylcholine infusion [64] suggesting that only the nerve signal was dysfunctional in that model. Several therapeutic categories have been analyzed from the perspective of some agents of the class (e.g., immunosuppressants and antipsychotics) producing adiposity as a side effect [67,68]. We suggest that such drugs may be acting by interfering with HISS production, release or action and resulting in AMIS. Therapeutics that inhibit MIS will have severely reduced usefulness.

## 10. Direct (HISS-Independent) Insulin Action and Adiposity

Relating adiposity to insulin or HISS sensitivity is hampered by the fact that the sensitivity is assessed at one time point but the adiposity represents a gradual lifetime accumulation of fat. Thus, a delay is anticipated between the detection of HISS-dependent insulin resistance (HDIR) and its effect on fat accumulation. On the other hand, if it is the fat that causes HDIR, there should be no such delay. 

Increasing the severity of AMIS is associated with earlier onset of adiposity and more rapid reduction in direct insulin action. Increased HISS action is associated with delayed onset of obesity and maintained direct insulin action. Reduced HISS-independent insulin action is not detectable in acute models of AMIS, including up to 9 weeks of sucrose supplement. However as the AMIS models become more severe or develop with time (age to 52 weeks with sucrose supplement), the direct insulin action becomes significantly impaired. HISS action decreases sooner and faster than the decrease in insulin action. HISS action decreases at approximately 4 times the rate of the decrease in the direct, or HISS-independent, insulin action. The decrease in HISS-independent insulin action is suggested to be secondary to HDIR-dependent adiposity [48].

## 11. Fatty Liver

The parallel increase in obesity and diabetes has led to the proposal that accumulated ectopic fat, including that in the liver, leads to peripheral insulin resistance and diabetes (e.g., reviews [69,70,71]). However, this paradigm does not offer a mechanistic explanation for the cause of elevated hepatic triglyceride nor for how a fatty liver would result in peripheral insulin resistance. In our models, the whole body fat content rose in direct and strong linear correlation with the fat pads that drain into the portal vein as well as those that do not. The perinephric fat pad is the easiest to dissect and has the highest correlation. The weight of the fat pads as % total body fat remained similar over the full range of adiposity [48]. However, there is clearly some degree of lipo-specificity in some organs like the liver and heart. Elevation in hepatic triglyceride levels did not occur early in the AMIS syndrome, but appeared significant only in the most severe model (52 weeks of aging with a sucrose supplemented diet, unpublished observation). Hepatic lipidemia does not cause peripheral insulin resistance but represents a relatively advanced stage of the AMIS syndrome.

## 12. Fatty Heart

Cardiac dysfunction occurs early in these models [72]. Rats aged 9, 26 and 52 weeks showed strong age-related cardiovascular deterioration, assessed partly on* in vivo* left ventricular dynamic pressure-volume loop analysis. Progressive reduction was seen in the rate of systolic and diastolic force development, ejection fraction, cardiac output index, and ventricular elasticity. Age-related elevations occurred in end diastolic ventricular pressure, arterial blood pressure and total peripheral vascular resistance (reduced conductance). These dysfunctions correlated with the degree of AMIS and were made worse by a sucrose-supplemented diet and were protected by Samec.

However cardiac lipid levels showed small variation and did not change significantly in any models. The heart is one of the selective tissue targets for HISS action, with 35% of the cardiac glucose uptake being accounted for by HISS action [19]. Absence of HISS action results in cardiac dysfunction by unknown mechanism, but the dysfunctions are not caused by fat accumulation.

## 13. Exercise

The effect of exercise, diet and antioxidants on the AMIS syndrome in healthy, aging and prediabetic rats has been reviewed [10]. Aging (young to middle age) results in AMIS which progresses over the tested period of 9, 14 and 26 weeks [73]. The distance voluntarily run increased daily for 7 days for all ages. HISS improvement per distance run was greatest in the oldest group, although they ran the least. HISS action correlated with the extent of running at all age groups. Combining the data from all 3 age groups with and without one week of exercise showed correlation of absolute HISS action with fat pad mass. Insulin levels increased with age as HISS action decreased. Exercise reduced insulin levels in all age groups to or below levels in non-exercised young (9 weeks) rats.

The effect of 7 day voluntary running-wheel exercise in prediabetic rats with severe diet-induced AMIS was determined by measuring insulin- and HISS action in the exercised rats and comparing them with the non-exercised controls. Voluntary exercise reversed insulin resistance, caused by a high fat or sucrose diet, through restoration of the HISS action. HISS action was increased and insulin levels were subsequently reduced by exercise. The direct insulin action was not changed by either diet or exercise. The metabolic improvements and reduced adiposity correlated with the extent of reversal of HISS action induced by exercise. 

Exercise improves insulin sensitivity in diet-induced insulin resistance primarily by restoration of HISS-mediated glucose uptake. Exercise shifts nutrient energy storage from lipids to glycogen through elevating HISS secretion in response to insulin. The interaction of antioxidant supplements on the metabolic benefits of exercise has been controversial, however the antioxidants used in clinical trials were not balanced to act throughout the cell. The balanced antioxidant, Samec, had the same protective effect on AMIS as exercise and did not interfere with the effect of exercise [30].

## 14. SAMEC and Free Radical Quenching

A balanced, synergistic antioxidant cocktail, composed of *S*-adenosyl methionine, vitamin E and vitamin C (Samec), provided protection of HISS action in chemically-induced liver damage, and has subsequently been used as one tool to alter MIS. Samec in daily chow conferred protection against the AMIS that developed with age, and blocked the effect of the sucrose diet on AMIS in old rats by preventing the decline in HISS action caused by age and diet (Figure 8). Each group showed strong correlations of AMIS (reduced HISS action) with adiposity (Figure 9). 

The strong negative effect of the sucrose supplemented diet is not related to caloric load as rats given an isocaloric solid food did not develop AMIS, whereas the rats that had sucrose in the solid food or liquid form had severe AMIS by 2 weeks [57]. Chow and sucrose consumption were not different in the chronic groups that had Samec, showing that the degree of adiposity is not necessarily related to overconsumption of calories, and in these studies it is related to reduction in HISS action and reduced MIS. 

## 15. Human Data

The results of a clinical study by Petersen* et al.* [74] are consistent with the HISS hypothesis. They showed that in lean insulin-resistant subjects skeletal muscle insulin resistance predates hepatic insulin resistance and was associated with a 60% increase in plasma triglyceride. They also showed that muscle glycogen synthesis was reduced by ~60% and suggested that abdominal obesity develops later in the course of the metabolic syndrome, and that it is likely a consequence of insulin resistance in skeletal muscle. The mechanism of the initiating skeletal muscle insulin resistance was not suggested but is compatible with HISS-dependent insulin resistance and the AMIS syndrome. 

Testing of the HISS hypothesis in humans confirms the data from animal studies. The first demonstration of MIS in humans was shown by Patarrao* et al.* 2008 [15]. The RIST index (amount of glucose infusion required to hold the glycemic baseline constant after a bolus injection of 50 mU/kg insulin) increased from a 24 hours fasted response of 215 to a postprandial response of 681 mg/kg, a MIS of 232% (Figure 1). Atropine, in submaximal dose, reduced the fed RIST index by 56%. 

Although not able to determine causality, the association of mild obesity (BMI 22.7 *versus* 27.7) with AMIS was confirmed by Patarrao* et al.* 2012 [16]. Twenty-four hour fasting glucose levels were not elevated but insulin, triglycerides and LDL cholesterol were all elevated in the overweight group. In lean and overweight subjects the response to insulin in the 24 hours fasted state was not different (215* vs.* 178 mg/kg, lean* vs.* overweight), showing that the direct effect of insulin, in the absence of HISS was not significantly altered in the fasted state. However 100 minutes after consumption of a test meal, the RIST index rose to 681 (232%) in lean and only 389 (119%) in the overweight group [14,15,16]. 

## 16. Conclusions 

The absence of HISS action shifts the balance of postprandial storage of nutrient energy primarily to lipid, and is first detected as elevations in postprandial glucose, insulin and triglycerides. Progressive adiposity develops with whole body fat content and fat pad weight increasing in parallel well before fasting glucose levels are elevated. In contrast to the generalized storage in fat depots, fat accumulation in the liver occurs in the late prediabetic or early diabetic stage of the AMIS syndrome. Treatment directed to preservation of MIS using a balanced antioxidant cocktail, resulted in reductions in postprandial insulin, glucose and lipids, which correlated with preserved HISS action and prevention of adiposity at all fat depots. The heart is a target of HISS action, and AMIS correlated with cardiovascular dysfunctions in the absence of accumulated lipids. AMIS can be diagnosed, based on the response to a mixed meal, can be prevented by supplementation with a balanced, synergistic antioxidant combination and can be reversed with one pre-meal dose of drugs to mimic the feeding signals. Hopefully this paradigm will be further investigated and applied to human health.
